# Microbial Nano-Factories: Synthesis and Biomedical Applications

**DOI:** 10.3389/fchem.2021.626834

**Published:** 2021-04-16

**Authors:** Shubhrima Ghosh, Razi Ahmad, Md. Zeyaullah, Sunil Kumar Khare

**Affiliations:** ^1^Enzyme and Microbial Biochemistry Laboratory, Department of Chemistry, Indian Institute of Technology Delhi, New Delhi, India; ^2^Department of Basic Medical Science, College of Applied Medical Science, King Khalid University (KKU), Khamis Mushait, Abha, Saudi Arabia

**Keywords:** nanoparticles, microbial, synthesis, biogenic, metals, biocompatible, biomaterial, therapeutic

## Abstract

In the recent times, nanomaterials have emerged in the field of biology, medicine, electronics, and agriculture due to their immense applications. Owing to their nanoscale sizes, they present large surface/volume ratio, characteristic structures, and similar dimensions to biomolecules resulting in unique properties for biomedical applications. The chemical and physical methods to synthesize nanoparticles have their own limitations which can be overcome using biological methods for the synthesis. Moreover, through the biogenic synthesis route, the usage of microorganisms has offered a reliable, sustainable, safe, and environmental friendly technique for nanosynthesis. Bacterial, algal, fungal, and yeast cells are known to transport metals from their environment and convert them to elemental nanoparticle forms which are either accumulated or secreted. Additionally, robust nanocarriers have also been developed using viruses. In order to prevent aggregation and promote stabilization of the nanoparticles, capping agents are often secreted during biosynthesis. Microbial nanoparticles find biomedical applications in rapid diagnostics, imaging, biopharmaceuticals, drug delivery systems, antimicrobials, biomaterials for tissue regeneration as well as biosensors. The major challenges in therapeutic applications of microbial nanoparticles include biocompatibility, bioavailability, stability, degradation in the gastro-intestinal tract, and immune response. Thus, the current review article is focused on the microbe-mediated synthesis of various nanoparticles, the different microbial strains explored for such synthesis along with their current and future biomedical applications.

## Introduction

Nanoparticles have found increasing industrial and biomedical applications in recent times. Particles within the size of 10–1,000 nm are considered as nanoparticles (Arshad, 2017). However, in general for most applications, <100 nm is deemed to be effective for applications due to easier penetration and similar sizes to biomolecules. The smaller size of nanomaterials provide myriad research opportunities for biologists. Owing to their dimensions matching the scale of biomolecules, nanomaterials have the ability to interact with complex biological systems in unique ways. This rapidly expanding field has allowed for the design and development of multifunctional nanoparticles to diagnose target and treat diseases such as cancer (Sardar et al., 2014; Pastorino et al., 2019). Nanoscale molecules, components, and devices are essentially of the same scale as biological entities and can easily cross the blood-tissue barriers. New approaches such as drug delivery through nanocarriers are being used for targeted and controlled delivery to the specific site. They help in improving drug efficacy and decrease the drug toxicity in disease therapy (Blanco et al., 2015; Pastorino et al., 2019; Ahmad et al., 2021). Further, nanocarriers interact with the biomolecules on the cell surface and within the cell in ways that do not alter these molecules' biochemical properties and behavior (Pastorino et al., 2019; Gao et al., 2020; Stillman et al., 2020). Such ready access to a living cell's interior allows remarkable advantages on the clinical and basic research frontiers. These days, with unique optical properties such as fluorescence and surface plasmon resonance (SPR), nanomaterials are achieving increasing attention in biomedical applications (Wang et al., 2007; Boisselier and Astruc, 2009; Aminabad et al., 2019; Elahi et al., 2019) especially in developing optics-based analytical techniques used for bioimaging (Xia, 2008; Chisanga et al., 2019) and biosensing (Kumar et al., 2019; Celiksoy et al., 2020; Noori et al., 2020). For such biomedical applications, a metal surface's biocompatibility is a key consideration and metal nanoparticles synthesized using biological systems, provide metals ions with high biocompatibility.

**Figure 1:**
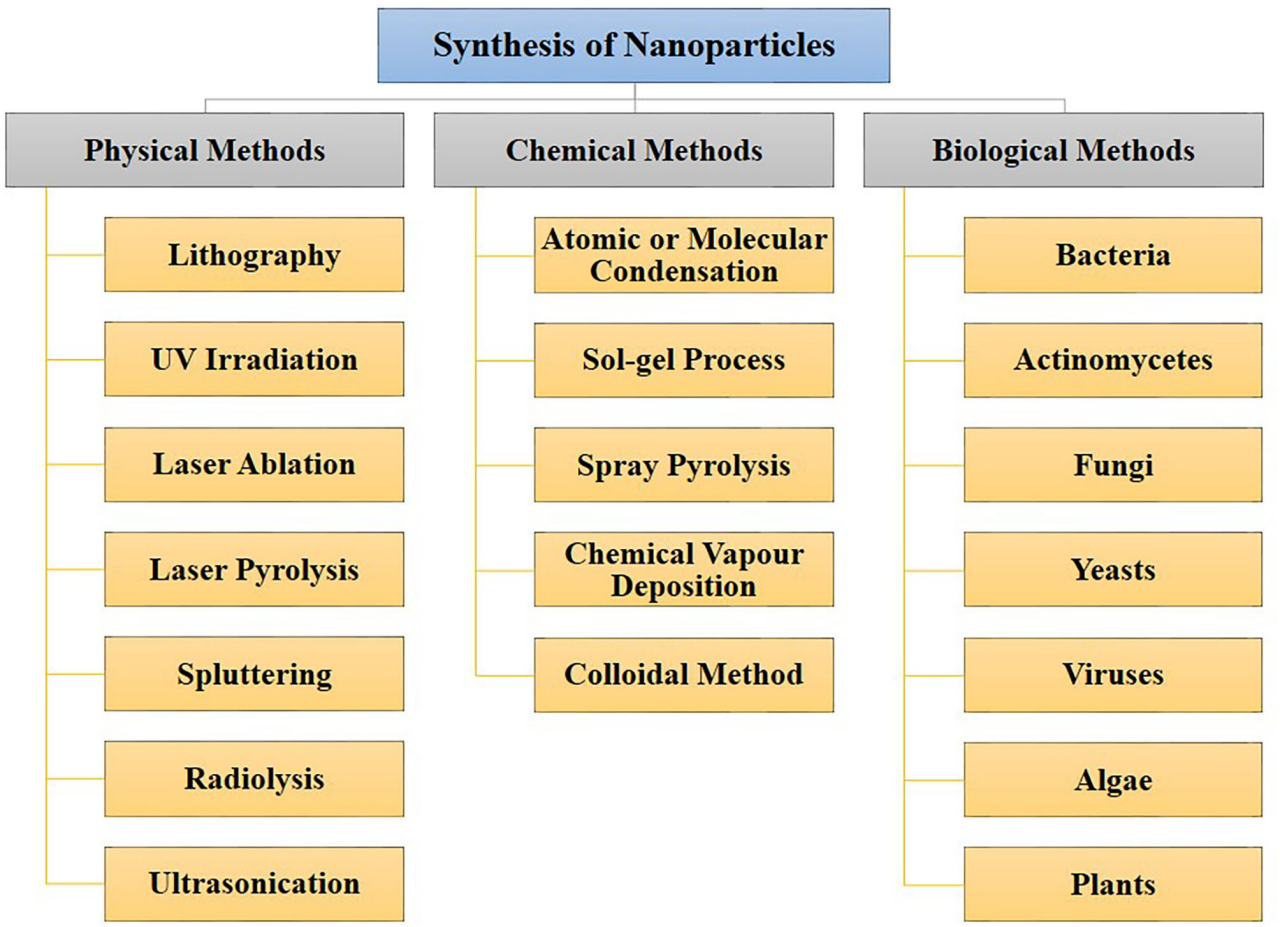
Different approaches for nanoparticles synthesis. Nanoparticles can be synthesized through physical, chemical, and biological routes.

Various nanoparticle synthesis methods include physical, chemical, and biological routes (Chen and Mao, 2007; Ahmad et al., 2015; Khatoon et al., 2015; Mazumder et al., 2016; Abdulla et al., 2021). The different physical, chemical, and biological methods of nano-synthesis are depicted in Figure 1. Green synthesis approaches such as biological means provide a sustainable, economical and less harsh nanoparticle synthesis method compared to chemical or physical methods. Moreover, biological synthesis offers control over size and shape for required applications. This is now well-known that many organisms can produce inorganic materials either intra or extracellularly (Senapati et al., 2004). Organisms such as bacteria, actinomycetes, fungi, yeasts, viruses, and algae are being explored as reducing or stabilizing agents to synthesize metal nanoparticles such as gold, silver, copper, cadmium, platinum, palladium, titanium, and zinc, which find uses in numerous industrial and biomedical application. Hence, the current review article is focused on the microbial-mediated synthesis of various nanoparticles and their applications in multiple sectors, with a particular focus on the biomedical and pharmaceutical industry.

## Synthesis of Nanoparticles by Microbial Strains

**Figure 2:**
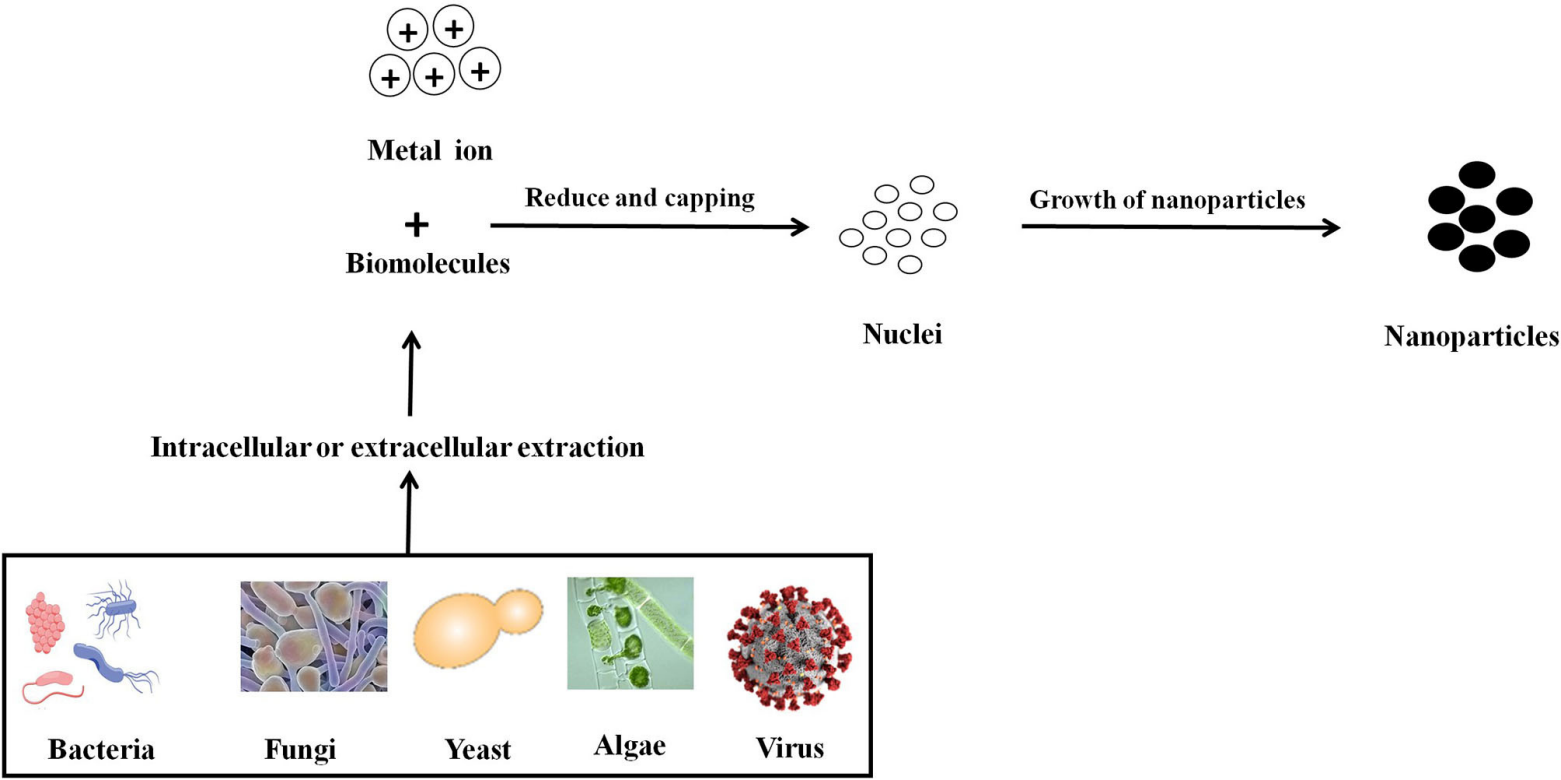
Mechanistic representation of the synthesis of nanoparticle by microbes. Formation of nanoparticles by microbes involves metal capture, enzymatic reduction, and capping. Metal ions are first trapped on the surface or inside of the microbial cells and then reduced to nanoparticles in the presence of enzymes. The enzyme serves as the nucleation site, providing electrons to the metal for its reduction. Microorganisms can impact mineral formation through the production of organic polymers, which can impact nucleation by favoring (or inhibiting) the stabilization of the very first mineral seeds.

There are three primary approaches to the synthesis of nanoparticles, namely physical, chemical, and biological. These three approaches of nanoparticles synthesis belong to either the top-down or bottom-up methods. The top-down approach involves the mechanical method of reducing size by gradually breaking down the bulk materials into the nanoscale structure. On the other hand, the bottom-up method is based on the assembly of atoms or molecules in the nanoscale range into the molecular structure. The bottom-up method depends on the nanoparticles' chemical and biological synthesis while top-down approaches generally refer to the physical or chemical route (Gan and Li, 2012; Lombardo et al., 2020). UV irradiation, sonochemistry, radiolysis, laser ablation, are physical methods to synthesize metallic nanoparticles (Kundu et al., 2008; Mohamed and Abu-Dief, 2018; Maric et al., 2019; Sadrolhosseini et al., 2019; Silva et al., 2019; Amulya et al., 2020). These methodologies have their limitations. While physical and chemical methods have successfully generated nanoparticles of high purity and desired size, these processes are typically costly and require toxic chemicals. The chemical synthesis process may lead to the existence of certain toxic chemical species becoming adsorbed on the surface of nanoparticles, which may lead to detrimental effects in medical applications; these nanoparticles may also have direct interaction with the human body, where the related toxicity becomes important. Thus, one of nanotechnology's primary objectives is to establish an eco-friendly production process that can provide low toxicity nanoparticles. Several investigators have focused their interest on biological methods of synthesizing metal nanoparticles to achieve this objective, as these are fast, cost-effective and eco-friendly. For this reason, the biological synthesis of nanoparticles includes a vast range of species in nature, such as viruses, bacteria, fungi, algae, plants (using their enzymes, proteins, DNA, lipids, and carbohydrates, etc.). Bacteria that reduce metals are found environmental-friendly catalysts for bioremediation as well as materials synthesis. In fact, microbes may help in the synthesis of diverse metal oxides through respiration processes (Kim et al., 2018). Electrons can be moved from reduced organic to oxidized inorganic compounds through microbial dissimilatory anaerobic respiration, thus promoting the formation of crystal/nanoparticles along with bioremediation processes. It is well-documented that the genus *Shewanella* are able to do the oxidation of organic acids as electron donors and reduction of inorganic metals as electron acceptors (Heidelberg et al., 2002; Harris et al., 2018). Further, the mechanism for detoxifying the immediate cell environment has been developed by microorganisms such as bacteria by reducing toxic metal species into metal nanoparticles (Deplanche and Macaskie, 2008; Murray et al., 2017). Also, biomolecules secreted by bacteria was used as capping as well as stabilizing agents of nanoparticles synthesis. The nanoparticle synthesis by the microbial process is depicted in Figure 2. The nanoparticles are usually formed following the way where metal ions are first trapped on the surface or inside of the microbial cells. The trapped metal ions are then reduced to nanoparticles in the presence of enzymes. In general, microorganisms impact the mineral formation in two distinct ways. They can modify the composition of the solution so that it becomes supersaturated or more supersaturated than it previously was with respect to a specific phase. A second means by which microorganisms can impact mineral formation is through the production of organic polymers, which can impact nucleation by favoring (or inhibiting) the stabilization of the very first mineral seeds. Microbes, which are regarded as potent eco-friendly green nanofactories, have the potential to control the size and shape of biological nanoparticles. Even though plant-extract based nanoparticle synthesis is a well-known biological nanosynthesis platform, nanoparticles synthesized this way may become polydisperse in nature due to the presence of phytochemicals as well as have difference in yield due to seasonal variations (Mishra A. et al., 2013; Mishra et al., 2016; Ovais et al., 2018; Sadaf et al., 2020; Ahmad et al., 2021). Thus, these are the distinct advantages pertaining to the synthesis of nanoparticles by microbes as compared to plants. Therefore, many microorganisms are considered to be potential candidates for synthesis of nanoparticles (Priyadarshini et al., 2013). The list of microorganisms used for the synthesis of nanoparticles is summarized in Table 1.

**Table 1:** Microbial mediated synthesis of nanoparticles.

	**Nanoparticles**	**Size**	**Application**	**References**
**Bacteria**				
*Bacillus subtilis*	TiO_2_	10–30 nm	Photocatalytic effect on aquatic biofilm	Dhandapani et al., 2012
*Lactobacillus* sp.	TiO_2_	50–100 nm	Antibacterial activity	Ahmad et al., 2014
*Lactobacillus* sp.	TiO_2_	50–100 nm	Immobilization and refolding of enzyme	Ahmad et al., 2013
*Escherichia coli*	Ag	5–50 nm	Antimicrobial Activity	Saeed et al., 2020
*Exiguobacterium aurantiacumm*	Ag	5–50 nm	Antimicrobial Activity	Saeed et al., 2020
*Brevundimonas diminuta*	Ag	5–50 nm	Antimicrobial Activity	Saeed et al., 2020
Thermophilic *Bacillus* sp. AZ1	Ag	9–32 nm	Antimicrobial Activity	Deljou and Goudarzi, 2016
*Gordonia amicalis*	Ag	5–25 nm	Antioxidant scavenging activity	Sowani et al., 2016
*Lactobacillus acidophilus*	Ag	45–60 nm	Genomic toxicity	Namasivayam et al., 2010
*Acinetobacter* sp. SW30	Au	15–40 nm		Wadhwani et al., 2016
*Lactobacillus kimchicus* DCY51	Au	5–30 nm	Antioxidant activity	Markus et al., 2016
*Paracoccus haeundaensis* BC74171	Au	20.93 ± 3.46 nm	Antioxidant activity and antiproliferative effect	Patil et al., 2019
*Micrococcus yunnanensis*	Au	53.8 nm	Antibacterial, Anticancer	Jafari et al., 2018
*Mycobacterium* sp.	Au	5–55 nm	Anticancer	Camas et al., 2018
*Lactobacillus* sp.	CdS	2.5–5.5 nm		Prasad and Jha, 2010
*Aeromonas hydrophila*	ZnO	57.7 nm	Antimicrobial activity against *Pseudomonas aeruginosa* and *Aspergillus flavus*	Jayaseelan et al., 2012
*Lactobacillus plantarum*	ZnO	7–19 nm		Selvarajan and Mohanasrinivasan, 2013
*Lactobacillus sporogenes*	ZnO	145.70 nm	Antimicrobial activity	Mishra M. et al., 2013
*Bacillus subtilis*	Fe_3_O_4_	60–80 nm		Sundaram et al., 2012
*Lactobacillus fermentum*	Iron oxide	10–15 nm		Park et al., 2014
*Thermoanaerobacter ethanolicus*	Magnetite	35–65 nm		Yeary et al., 2005
*Shewanella loihica*	Cu	10–16 nm	Antibacterial activity	Lv et al., 2018
*Bacillus licheniformis*	cadmium sulfide	20–40 nm	Antibacterial activity	Shivashankarappa and Sanjay, 2015
*Serratia nematodiphila*	zinc sulfide	80 ± 10 nm	Antibacterial activity	Malarkodi and Annadurai, 2013
*Idiomarina* sp. strain PR58-8	Lead(IV) Sulfide	6–10 nm	Bioimaging	Srivastava and Kowshik, 2017
*Bacillus* sp.	Selenium nanoparticles	80–220 nm	Antioxidant and cytotoxic effect	Forootanfar et al., 2014
*Pantoea agglomerans*	Selenium nanoparticles	90–110 nm	Antioxidant activity	Torres et al., 2012
**Actinomycetes**				
*Rhodococcus* sp.(Actinomycete)	Au	5–15 nm		Ahmad et al., 2003b
*Gordonia amarae*	Au	15–40 nm	Application in rapid sensing of copper ions	Bennur et al., 2016
*Gordonia amicalis*	Au	5–25 nm	Antioxidant scavenging activity	Sowani et al., 2016
*Streptomycetes viridogens* HM10	Au	18–20 nm	Antibacterial activity	Balagurunathan et al., 2011
*Actinomycetes* sp.	Ag	10–20 nm	Antibacterial activity	Abdeen et al., 2014
Marine Isolate *Streptomyces albidoflavus*	Ag	10–40 nm		Prakasham et al., 2012
*Streptomyces* sp. LK3	Ag	5 nm	Acaricidal activity against *Rhipicephalus microplus* and *Haemaphysalis bispinosa*	Karthik et al., 2014
*Streptomyces* sp. JAR1	Ag	60–70 nm	Antimicrobial activity	Chauhan et al., 2013
*Nocardiopsis* sp. MBRC-1	Ag	45 nm	Antimicrobial activity	Manivasagan et al., 2013
Actinomycetes	Ag	5–50 nm	Antibacterial activity	Narasimha et al., 2013
*Streptomyces* sp. VITPK1	Ag	20–45 nm	Anticandidal activity	Sanjenbam et al., 2014
Marine endophytic actinomycetes	Cu	Nanorange size	Antibacterial efficacy	Rasool and Hemalatha, 2017
**Fungus**				
*Penicillium* sp.	Ag	25–30 nm	Antibacterial	Singh et al., 2014
*Neurospora crassa*	Ag	3–50 nm		Castro-Longoria et al., 2011
*Verticillium* sp.	Ag	25 ± 12 nm	Antimicrobial activity	Mukherjee et al., 2001
*Trichoderma longibrachiatum*	Ag	10 nm	Antifungal against phyto-pathogenic fungi	Elamawi et al., 2018
*Penicillium oxalicum*	Ag	60–80 nm	Antibacterial activity	Feroze et al., 2020
*Aspergillus niger*	Ag	13.2–646.8 nm	Antifungal effect	Gursoy, 2020
*Penicillium janthinellum DJP06*	Ag	1–30 nm		Pareek et al., 2020
*Cladosporium perangustum*	Ag	30–40 nm	Antioxidant, anticancer, and nano-toxicological study	Govindappa et al., 2020
*Macrophomina phaseolina*	Ag	5–40 nm	Antimicrobial properties	Chowdhury et al., 2014
*Neurospora crassa*	Au	3–100 nm		Castro-Longoria et al., 2011
*Trichoderma harzianum*	Au	32–44 nm	Antibacterial activity	Tripathi et al., 2018
*Morchella esculenta*	Au	16.51 nm	Antimicrobial activity and cytotoxic activity	Acay, 2020
*Cladosporium* sp.	Au	5–10 nm	Photodegradation, *in vitro* anticancer activity and *in vivo* antitumor studies	Munawer et al., 2020
*Penicillium janthinellum DJP06*	Au	1–40 nm		Pareek et al., 2020
*Neurospora crassa*	bimetallic Au/Ag	3–110 nm		Castro-Longoria et al., 2011
Coriolus versicolor	CdS	100–200 nm,		Sanghi and Verma, 2009
Thermophilic fungus *Humicola* sp.	CeO_2_	12–20 nm		Khan and Ahmad, 2013
*Aspergillus niger*	ZnO	53–69 nm	Antibacterial activity	Kalpana et al., 2018
*C. geniculatus*	ZnO	2–6 nm		Kadam et al., 2019
*Agaricus bisporus*	ZnS	2.9 nm		Senapati et al., 2015
*Fusarium oxysporum*	ZnS	~38 nm		Mirzadeh et al., 2013
*Penicillium chrysogenum*	Pt	5–40 nm	Cytotoxicity	Subramaniyan et al., 2018
*Aspergillus flavus*	TiO_2_	62–74 nm	Antimicrobial activity	Rajakumar et al., 2012
**Yeast**				
*Yarrowia lipolytica* (NCYC 789)	Ag	2–5 nm	Activity against *E. coli, S. aureus*	Apte et al., 2013
yeast strain MKY3	Ag	2–5 nm		Kowshik et al., 2002
*Yarrowia lipolytica* DSM 3286	Ag	12.4 ± 5.22 nm	Antibacterial activity	Bolbanabad et al., 2020
*Candida guilliermondii*	Ag	10–20 nm	Antimicrobial activity	Mishra et al., 2011
*Saccharomyces boulardii*	Ag	3–10 nm	Anticancer activity	Kaler et al., 2013
*Kluyveromyces marxianus*	Ag	3–12 nm	Antimicrobial agent	Ashour, 2014
*Candida utilis* 22	Ag	6–20 nm	Antimicrobial agent	Ashour, 2014
*Candida utilis*	Ag	20–80 nm	Antibacterial activity against pathogenic organisms	Waghmare et al., 2015
*Saccharomyces cerevisiae*	Ag	10–60 nm	Antimicrobial effect	Sowbarnika et al., 2018
*Candida glabrata*	Ag	2–15 nm	Antibacterial and antifungal	Jalal et al., 2018
*Rhodotorula glutinis*	Ag	15.45 ± 7.94 nm	Antifungal, catalytic and cytotoxicity activities	Cunha et al., 2018
*Rhodotorula mucilaginosa*	Ag	13.70 ± 8.21 nm	Antifungal, catalytic and cytotoxicity activities	Cunha et al., 2018
*Candida guilliermondii*	Au	50–70 nm	Antimicrobial activity	Mishra et al., 2011
*Yarrowia lipolytica* NCIM	Au	15 nm		Agnihotri et al., 2009
*Magnusiomyces ingens* LH-F1	Au	10–80 nm	Catalytic activities for the reduction of nitrophenols	Zhang et al., 2016
*Saccharomyces cerevisiae*	CdS	3.75 nm		Prasad and Jha, 2010
*Candida albicans*	CdS	50–60 nm	Bactericidal potential against *Salmonella typhi* and *Staphylococcus aureus*	Kumar et al., 2019
Baker's yeast	TiO_2_	6.7 ± 2.2nm	Antibacterial activity	Peiris et al., 2018
*Saccharomyces cerevisiae*	TiO_2_	12 nm		Jha et al., 2009a
Baker's yeast	Fe_2_O_3_	2–10 nm	Detection H_2_O_2_ and Glucose	Mishra et al., 2015
*Saccharomyces cerevisiae*	Sb_2_O_3_	100 nm		Jha et al., 2009b
*Saccharomyces cerevisiae*	Amorphous iron phosphate	50–200 nm		He et al., 2009
**Alga**				
*Neochloris oleoabundans*	Ag	40 nm	Antibacterial	Bao and Lan, 2018
*Enteromorpha compressa*	Ag	4–24 nm	Antimicrobial, Anticancer	Ramkumar et al., 2017
*Nostoc linckia*	Ag	5–60 nm	Antibacterial	Vanlalveni et al., 2018
*Leptolyngbya*	Ag	5–50 nm	Antibacterial, Anticancer	Zada et al., 2018
*Spyridia fusiformis*	Ag	5–50 nm	Antibacterial	Murugesan et al., 2017
*Chaetomorpha linum*	Ag	70–80 nm	Efficient anticancer agent	Acharya et al., 2021
*Chlorella ellipsoidea*	Ag	220.8 ± 31.3 nm	Photophysical, catalytic, and antibacterial activity	Borah et al., 2020
*Amphiroa rigida*	Ag	25 nm	Antibacterial, cytotoxicity, and larvicidal efficiency	Gopu et al., 2020
*Ulva armoricana* sp.	Ag	33 nm	Bactericidal activity	Massironi et al., 2019
*Spirulina platensis*	Au	15.60–77.13 nm	Antiviral activity	El-Sheekh et al., 2020
*Sargassum cymosum*	Au	7 and 20 nm		Costa et al., 2020
*Tetraselmis kochinensis*	Au	5–35 nm		Senapati et al., 2012
*Stephanopyxis turris*	Au	10–30 nm		Pytlik et al., 2017
*Galaxaura elongate*	Au	3.85–77 nm	Antibacterial	Abdel-Raouf et al., 2017
*Cystoseira baccata*	Au	8.4 nm	Anticancer	Gonzalez-Ballesteros et al., 2017
*Spirulina platensis*	Pd	10–20 nm	Adsorbent	Sayadi et al., 2018
*Chlorella vulgaris*	Pd	5–20 nm		Arsiya et al., 2017
*Sargassum wightii*	ZrO_2_	18 nm	Antibacterial	Kumaresan et al., 2018
*Chlorella pyrenoidosa*	CdSe QD	4–5 nm	Imatinib sensing	Zhang Z. et al., 2018

### Nanoparticle Synthesis by Bacteria

Production of reduced metal ions by microbes arises from their remarkable ability to adapt themselves to conditions of environmental stress (Kulkarni et al., 2015). Therefore, supernatants of various bacteria such as *Pseudomonas proteolytic, Pseudomonas meridiana, Pseudomonas Antarctica, Arthrobacter gangotriensis*, and *Arthrobacter kerguelensis* act as microbial cell factories finding applications as reducing agents in the synthesis of silver nanoparticles (Shaligram et al., 2009; Singh et al., 2015). Silver nanoparticles (AgNPs) synthesized by using *Bacillus brevis* have recently demonstrated remarkable antimicrobial properties against *Staphylococcus aureus* and *Salmonella typhi* multidrug-resistant strains (Saravanan et al., 2018). *Pseudomonas stutzeri* is another bacterial strain which has been found to accumulate AgNPs through an intracellular mechanism (Klaus et al., 1999). In *Bacillus* sp., silver nanoparticles have also been synthesized in intracellular periplasmic space (Pugazhenthiran et al., 2009). The organisms that reside in gold mines would be more able to resist soluble gold toxicity and efficiently produce gold nanoparticles (Srinath et al., 2018). When *Acinetobacter* sp. SW30 was incubated with different concentrations of gold chloride and different cell density, it showed enormous variation in the color of gold nanoparticles (AuNP) containing colloidal solution, suggesting variation in size and shape. Surprisingly, at the lowest cell density and HAuCl_4_ salt concentration, monodispersed spherical AuNP of size ~19 nm was observed, whereas cell number increase resulted in polyhedral AuNP (~39 nm) formation. Amino acids are implicated in the gold salt reduction, while amide groups assist in AuNP stabilization (Wadhwani et al., 2016). Also, inside the lactic acid bacteria cells, nanocrystals of silver, gold, and their alloys have been biosynthesized (Nair and Pradeep, 2002). In order to synthesize gold nanoparticles (AuNPs), two separate strains of *Pseudomonas aeruginosa* were used in one sample, producing AuNPs of different sizes (Husseiny et al., 2007). *Rhodopseudomonas capsulate* mediated extracellular synthesis of gold nanoparticles of various sizes and shapes was also reported. The strain was used to generate spherical (10–50 nm) and triangular plate (50–400 nm) AuNPs (He et al., 2007). ZnO nanoflowers were synthesized using *Serratia ureilytica* and further used on cotton fabrics to provide antimicrobial activities against *E. coli* and *S. aureus* (Dhandapani et al., 2014). *Lactobacillus plantarum* has also been reported to biosynthesize ZnO nanoparticles (Selvarajan and Mohanasrinivasan, 2013). The gram-negative bacterial strain *Aeromonas hydrophila* has been explored for the biosynthesis of ZnO nanoparticles with further antimicrobial applications (Jayaseelan et al., 2012). Triangular CuO nanoparticles have been developed using *Halomonas elongate* which displayed antimicrobial activity against *E. coli* and *S. aureus* (Rad et al., 2018). In another study, super paramagnetic iron oxide nanoparticles of about 29.3 nm dimensions were manufactured using *Bacillus cereus* strain. As an application, their anti-cancer effects were reported against the MCF-7 (breast cancer) and 3T3 (mouse fibroblast) cell lines in a dose-dependent manner (Fatemi et al., 2018). A rapid, convenient method for the synthesis of manganese and zinc nanoparticles by reducing manganese sulfate and zinc sulfate using *Streptomyces* sp. (intracellular route) has been reported. The scale of NPs for manganese and zinc was between 10 and 20 nm (Waghmare et al., 2011). *Bacillus amyloliquifaciens* strain KSU-109 produced surfactin, which helped in the synthesis of stable cadmium sulfide nanoparticles of average size of 3–4 nm (Singh et al., 2011). *Escherichia coli* E-30 and *Klebsiella pneumoniae* K-6 have been shown to synthesize cadmium sulfide nanoparticles with average size ranging from 3.2 to 44.9 nm and showed highest antimicrobial activity on *A. fumigatus, G. candidum, B. subtilis, S. aureus*, and *E. coli* strains (Abd Elsalam et al., 2018). *Serratia marcescens* mediated synthesized antimony sulfide nanoparticles were reported with size range <35 nm (Bahrami et al., 2012), while *Pseudomonas aeruginosa* ATCC 27853 mediated synthesis of selenium nanoparticles were reported with a size of 96 nm (Kora and Rastogi, 2016). Lead nanoparticles synthesized using *Cocos nucifera* were reported with 47 nm size and also showed good activity against *S. aureus* (Elango and Roopan, 2015). The bacterial strains isolated from Gabal El Sela in Eastern Dessert, Egypt have been used for the biosynthesis of uranium nanoparticles intracellularly with size ranging from 2.9 to 21.13 nm (Abostate et al., 2018).

Cyanobacteria are a phylum of photosynthetic bacteria widely explored for their capacity to synthesize nanoparticles due to the presence of bioactive components, which help in stabilizing and functionalizing the nanoparticles, resulting in fewer steps in synthesis. Their high-growth rate also facilitates higher biomass production to aid in nanosynthesis. In most cases, cell-free extracts of the cyanobacterial biomass are used for nanosynthesis. Aqueous extracts of the cyanobacterium *Oscillatoria limnetica* has been useful in synthesizing silver nanoparticles by reduction and further stabilizing them. The size of the nanoparticles was 3.30–17.97 nm and they showed anti-cancer and anti-microbial activity (Hamouda et al., 2019). A similar Ag-NPs synthesis by *Microchaete* sp. NCCU-342 was pursued using aqueous biomass extracts and spherical, polydispersed nanoparticles of 60–80 nm size were obtained (Husain et al., 2019). Silver nanoparticles synthesized from *Desertifilum* sp. (4.5–26 nm) showed antibacterial activity and cytotoxic effects against HepG2, MCF-7, and Caco-2 cancer cells (Hamida et al., 2020). Other cyanobacterial strains explored for nanoparticle synthesis include *Scytonema* sp., *Nostoc* sp., *Phormidium* sp. (Al Rashed et al., 2018). One interesting study used filamentous cyanobacterium, *Plectonema boryanum* (strain UTEX 485) biomass reacted with AgNO_3_. Silver nanoparticles were found to precipitate on the surface as well as inside of the cyanobacterium cell. Intracellular nanoparticles were found to be of the size (<10 nm), while that of extracellular ones exhibited size in the range of (1–200 nm) (Lengke et al., 2007a). *P. boryanum* is also reported to reduce gold (III)-chloride solutions to form Au nanoparticles intracellularly via formation of gold (I) sulfide (Lengke et al., 2006b); this species is also known to produce platinum and palladium NPs (Lengke et al., 2006a, 2007b). Thus, cyanobacteria present a promising platform for biogenic nanosynthesis with widespread applications.

### Nanoparticle Synthesis by Actinomycetes

Actinomycetes have gained significant attention because they are the least studied, but important for metal nanoparticle synthesis (Golinska et al., 2014). Actinomycetes are considered superior groups among microbial species of commercial importance due to the development of various bioactive components and extracellular enzymes through their saprophytic behavior (Kumar et al., 2008). For the biosynthesis and characterization of gold nanoparticles, only a few of the genera such as *Thermomonospora, Nocardia, Streptomyces*, and *Rhodococcus* have been identified from actinomycetes (El-Batal et al., 2015). *Streptomyces* species are considered to be the dominant biosynthesis contender (Zonooz et al., 2012). In actinomycetes, intracellular reduction of metal ions takes place on the surface of mycelia along with cytoplasmic membranes, leading to the formation of nanoparticles (Ahmad et al., 2003b). Some researchers suggested that the possible mechanism of intracellular synthesis of metal nanoparticles occurs by trapping Ag^+^ ions on cell surface, likely through electrostatic interactions between Ag^+^ and negatively charged groups of carboxylate in mycelial cell wall enzymes. Enzymes present in the cell wall leading to the formation of silver nuclei decrease the silver ions, subsequently expanding by further decrease and accumulation of Ag^+^ ions on these nuclei (Abdeen et al., 2014). A different mechanism for the intracellular synthesis of silver nanoparticles by using lactic acid bacteria was suggested by Sintubin et al. (2009). Furthermore, several other researchers have also documented the intracellular synthesis of metal nanoparticles utilizing actinomycetes strains (Usha et al., 2010; Balagurunathan et al., 2011; Prakasham et al., 2012; Sukanya et al., 2013).

### Nanoparticle Synthesis by Fungi

Another biogenic route of biosynthesis of various metal nanoparticles involves successful application of myco-nanotechnological approaches. Similar to bacteria/cyanobacteria, nanosynthesis may be extracellular or intracellular in nature. In the intracellular route, metal salts in the mycelia, which fungi can use, are converted into a less toxic form (Molnar et al., 2018; Rajeshkumar and Sivapriya, 2020). The use of fungal extracts involves extracellular biosynthesis (Zhao et al., 2018; Rajeshkumar and Sivapriya, 2020). In the biosynthesis of nanoparticles, fungi are comparatively more resourceful than bacteria due to many bioactive metabolites, high aggregation, and improved production (Castro-Longoria et al., 2011; Alghuthaymi et al., 2015). Several filamentous fungi have been reported to be capable in AuNP biosynthesis. In order to biosynthesize AuNPs, this study employed various methods. The authors suggested that fungal secreted compounds and media components could be used to stabilize the nanoparticles (Molnar et al., 2018; Guilger-Casagrande and de Lima, 2019). Three different fungal strains (namely *Fusarium oxysporum, Fusarium* sp., and *Aureobasidium pullulans*) were used by another group to biosynthesize the reported AuNPs. The authors suggested that biosynthesis happened inside fungal vacuoles, and that sugar reduction was involved in tailoring the shape of AuNPs. Additionally, fungus produced the secondary metabolite contain protein or biomolecules which act as capping as well as stabilizing agents (Zhang et al., 2011). Several *Fusarium oxysporum* strains have been used in another study to generate extracellular silver metal nanoparticles in the 20–50 nm range (Ahmad et al., 2003a). The metal ion reduction by nitrate-dependent reductase and extracellular shuttle quinone was confirmed by UV-Visible, fluorescence, and enzymatic activity analysis (Duran et al., 2005, 2007). Kumar and their groups formed *in vitro* silver nanoparticles (10–25 nm) stabilized in the presence of reduced cofactor nicotinamide adenine dinucleotide phosphate (NADPH) by a capping peptide using the nitrate reductase enzyme isolated from *Fusarium oxysporum*, along with phytochelatinin, and 4-hydroxyquinoline (Kumar et al., 2007). Another study indicated that the synthesis of monodispersed AgNPs of 9.4 nm size was mediated by *Rhizopus stolonifera* extracts, although condition optimization resulted in AgNPs of 2.86 nm (Abdelrahim et al., 2017). The extracellular synthesis of AgNPs utilizing *Candida glabrata* suggested strong antimicrobial activity (Jalal et al., 2018). ZnO nanoparticles mediated by *Aspergillus niger* indicated excellent antibacterial potential, while the Bismarck brown dye was also degraded by up to 90% (Kalpana et al., 2018). Cobalt oxide nanoparticles have recently been fabricated using *Aspergillus nidulans* (Vijayanandan and Balakrishnan, 2018). Biosynthesis of platinum nanoparticles of size range 100–180 nm from the *Fusarium oxysporum* fungus was documented (Riddin et al., 2006). The fungi *Verticillium* sp., *Fusarium oxysporum* sp., and *Aspergillus flavus* have shown the ability to produce nanoparticles either extracellularly or intracellularly (Mukherjee et al., 2002; Bhainsa and D'Souza, 2006). To create natural nanofactories, the change from bacteria to fungi has the added benefit that downstream biomass processing and handling can be much more straightforward.

### Nanoparticle Synthesis by Yeasts

Yeast strains of several genera are known to employ different mechanisms for nanoparticle synthesis resulting in significant variations in size, particle position, monodispersity, and other properties. One study found that glutathione (GSH) and two classes of metal-binding ligands-metallothioneins and phytochelatins (PC) were generated by detoxification mechanisms in yeast cells. These molecules have a role to play in deciding the mechanism for nanoparticle synthesis and stabilize the resulting complexes in most of the yeast species studied (Hulkoti and Taranath, 2014). Often as a resistance mechanism, yeast cells in the vicinity of toxic metals can change the absorbed metal ions into complex polymer compounds that are not toxic to the cell. Typically, these nanoparticles synthesized in the yeast are referred to as “semiconductor crystals” or “quantum semiconductor crystals” (Dameron et al., 1989). Yeasts cells are particularly well-known for their ability to synthesize semiconductor nanoparticles, particularly that of cadmium sulfide (CdS). There are reports on the production of other metal nanoparticles, particularly AgNPs, by yeasts, including *Pichia capsulata* (Subramanian et al., 2010), *Candida guilliermondii* (Mishra et al., 2011), *Saccharomyces boulardii* (Kaler et al., 2013), *Kluyveromyces marxianus* (Ashour, 2014), *Candida utilis* (Waghmare et al., 2015), *Candida lusitaniae* (Eugenio et al., 2016), *Saccharomyces cerevisiae* (Sowbarnika et al., 2018), *Candida glabrata* (Jalal et al., 2018), *Candida albicans* (Ananthi et al., 2018), *Rhodotorula glutinis*, and *Rhodotorula mucilaginosa* (Cunha et al., 2018). The silver-tolerant yeast strain MKY3 was used for the production of silver nanoparticles (Kowshik et al., 2002).

### Nanoparticle Synthesis by Algae

The use of algae for the biosynthesis of nanoparticles is also increasingly becoming common. In order to synthesize ZnO nanoparticles, *Sargassum muticum* was used and was reported to decrease angiogenesis in HepG2 cells in addition to apoptotic effects (Sanaeimehr et al., 2018). In the biosynthesis of AuNPs, *Sargassum crassifolium*, a macroalgae along with sea grass, has been utilized. Interestingly in this study, a blue shift in the UV absorption spectra was observed after increasing the concentration of *S. crassifolium*, which was attributed to a decreased size of the nanoparticles due to increased nucleation centers in the reductant (Maceda et al., 2018). CuO nanoparticles of around 7 nm dimensions have been synthesized biogenically using *Cystoseira trinodis* and reported to have improved antibacterial and antioxidant properties, along with methylene blue degradation potential (Gu et al., 2018). Using *Sargassum ilicifolium*, aluminum oxide nanoparticles with ~20 nm size were produced (Koopi and Buazar, 2018). Various algae strains, for example *Turbinaria conoides, Laminaria japonica, Acanthophora spicifera*, and *Sargassum tenerrimum* have been reported to synthesize gold nanoparticles (Ghodake and Lee, 2011; Swaminathan et al., 2011; Vijayaraghavan et al., 2011; Ramakrishna et al., 2016). Using *Spirulina platensis*, synthesis of novel core (Au)-shell (Ag) nanoparticles has also been investigated (Govindaraju et al., 2008).

### Nanoparticle Synthesis by Viruses

Viruses have emerged as promising candidates as nanoparticles for biomedical applications, owing to their biocompatibility, biodegradability, capacity of mass production, programmable scaffolds, and ease of genetic manipulation for desired characteristics. Viral bodies, themselves are naturally occurring nanoparticles due to their 20–500 nanometer dimensions. Their robustness along with ability to detect changes in the environment to release their genetic material has been exploited in biomedical applications. The major applications of viral nanoparticles has been in gene delivery, drug delivery, as vaccines/immunotherapeutics and in imaging and theranostics. Mostly mammalian viruses are used in gene delivery while bacteriophages and plant viruses have been explored for drug delivery, vaccines, and immunotherapeutics. Viral nanoparticles (VNPs) can also be tagged with several ligands for targeting, therapeutics or imaging agents for myriad biomedical applications (Steinmetz, 2010). A similar class of materials are virus-like particles (VLPs) derived from the protein coating of the viruses (Chung et al., 2020). These nanoparticles can be of bacteriophage, plant or animal viral origin and are dynamic, self-assembling moieties with symmetrical, monodisperse structures. Production of viral nanoparticles involve generation in a host body (whether a bacteria, animal, or plant), further chemical conjugation and tuning, followed by evaluation *in-vitro* and *in-vivo* (Steinmetz, 2010). A major consideration for using VNPs is regarding their toxicity, especially for human pathogens. Thus, bacteriophages and plant viruses are preferred, compared to mammalian viruses such as adenoviruses. Additionally, immunogenicity of the viral particle affects their accumulation in the tissue as well as clearance. Attachment to molecules such as PEG, often helps in shielding of specific biointeractions (Bruckman et al., 2008). Various VNPs and VLPs have been exploited to deliver chemotherapeutic drugs. VLPs modified with targeting peptide with a load of doxorubicin, cisplatin, and 5-fluorouracil were found to be effective in human hepatocellular carcinoma cells (Ashley et al., 2011). Tobacco-Mosaic Virus derived VNPs used to carry cisplatin have been used in platinum-resistant ovarian cancer cells (Franke et al., 2017). Bacteriophage fd based nanoparticles with peptides specifically targeting pathogenic bacteria such as *Staphylococcus aureus*; and loaded with antibiotic such as chloramphenicol have found better antibacterial action than chloramphenicol alone (Yacoby et al., 2006). Viral nanoparticles also find application as MRI contrast agents, having large rotational correlation times due to their rigid structures, which results in high relaxivity. Additionally, owing to their polyvalent nature, a high number of contrast agents such as gadolinium can be chelated to their interior or exterior surfaces (Steinmetz, 2010). Such nanoparticles have also been explored to develop vaccines against pathogens such as hepatitis B, HIV, and *Neospora caninum* (Oh and Han, 2020).

In addition, one important precaution to be emphasized relates to the handling of bacterial or viral strains that might be harmful or pathogenic to humans. Thus, in order to implement microorganism-mediated nanosynthesis on a large scale for commercial exploitation, utmost importance is to be given to associated biological safety issues as well.

## Biological Application of Microbial Synthesized Nanomaterials

**Figure 3:**
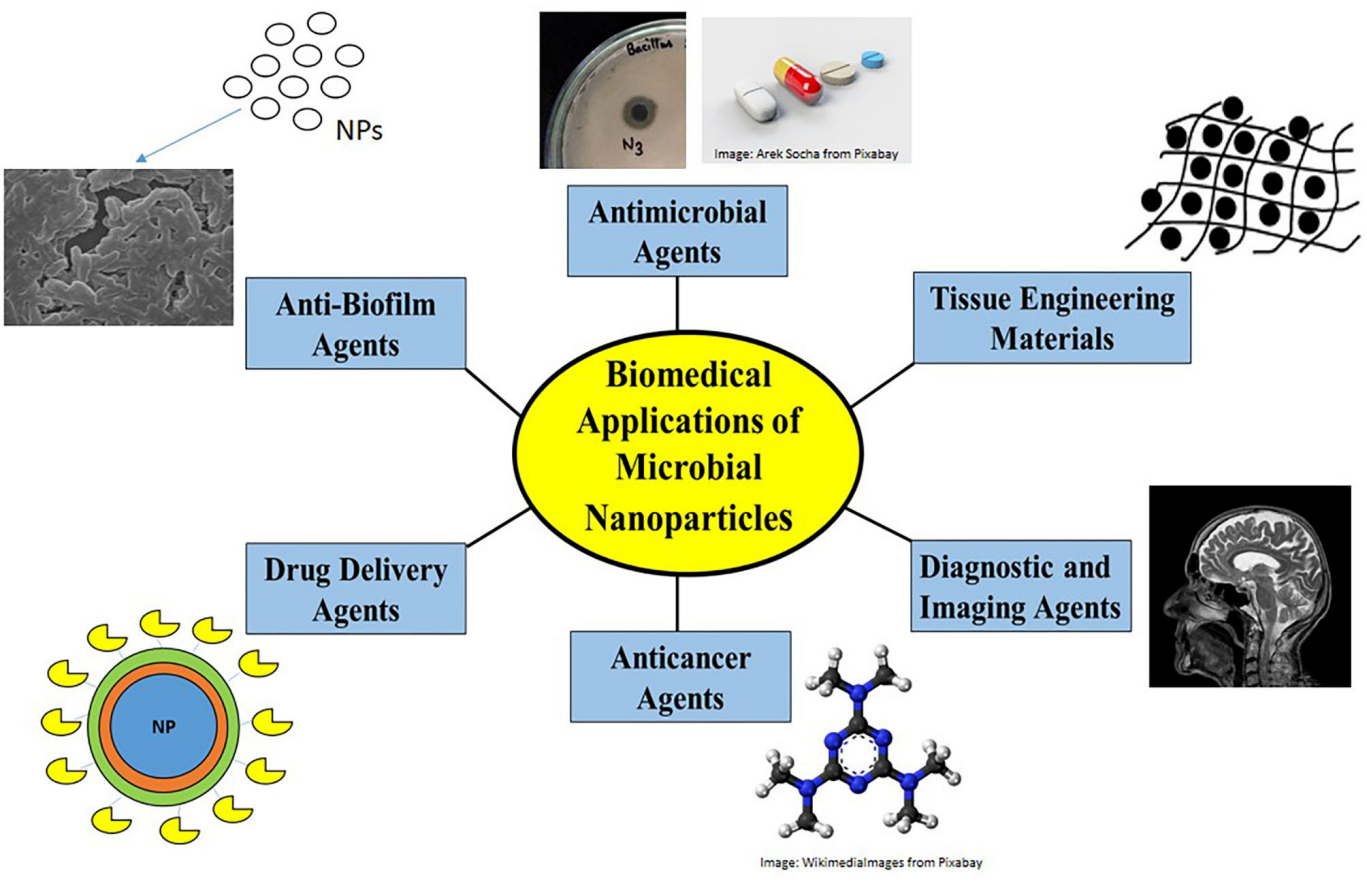
Biomedical applications of nanoparticles. Nanoparticles find biomedical applications as antimicrobial agents by disrupting membrane structure or by generating ROS, as anti-biofilm agents in preventing antimicrobial resistance, as drug-delivery agents to carry drug loads, as anti-cancer agents causing apoptosis, as diagnostic/imaging agents in MRI and biosensors and as anticoagulants/anthelmintics/tissue engineering materials.

Due to their controlled sizes, unique properties, biocompatible nature, non-toxicity, microbial nanoparticles find myriad biomedical applications. They have found major applications in the biomedical and pharmaceutical fields as antimicrobials, anti-biofilm agents, antioxidants, anti-cancer agents, and diagnostic or imaging agents, some of which are discussed here and shown in Figure 3.

### Antimicrobial Agents

In general, several metallic nanoparticles such as that of silver, copper, zinc, magnesium, gold, and titanium are known for their antimicrobial properties. The mode of antimicrobial action attributed to the nanoparticles include disruption of membrane structure, pore formation on the microbial cell wall, inhibition of biofilm formation or production of reactive oxygen species (ROS) in case of metal oxide nanoparticles (Busi and Rajkumari, 2019). The antimicrobial property is heavily dependent on the nanoparticle size and shape, with smaller, monodispersed nanoparticles (with resulting larger surface to volume ratio) displaying greater antimicrobial tendencies (Duran et al., 2010). The search for novel antimicrobial nanoparticles has been fuelled by the rise of multidrug resistance (MDR) phenotype among pathogenic strains. An important advantage of biogenic synthesis is the inherent presence of natural stabilizing or capping agents such as polysaccharides or proteins on the nanoparticle surface upon synthesis, which reduces post-production steps to a large extent. AuNPs synthesized using the culture supernatant of *Ochrobactrum rhizosphaerae* were found to be coated with glycolipoprotein, with potent antibiotic activity against *Vibrio cholerae*. In case of fungally synthesized nanoparticles, the capping agents are generally proteinaceous in nature. Example of Ag-NPs synthesized intracellularly by the mushroom fungus, *Schizophyllum commune* and that of by *Trichoderma viride* showed capping by proteins and exhibited antibacterial activity against strains such as *Bacillus subtilis, Pseudomonas* sp., *Trichophyton mentagrophytes, K. pneumonia, Trichophyton simii, Trichophyton rubrum, E. coli, B. subtilis*, and *Klebsiella planticola*, respectively (Chitra and Annadurai, 2013; Arun et al., 2014). The Silver nanoparticles generally act due to the release of Ag+ ions which can disrupt bacterial membranes as well as interfere with DNA and protein synthesis. Similarly, gold nanoparticles, due to their photocatalytic activity, can be developed in conjugation with photosensitizers for antimicrobial photodynamic therapy. On exposure to near Infrared radiation (NIR), the heat produced destroys the bacterial cell wall (Busi and Rajkumari, 2019). Often conjugation of traditional antibiotic moieties to nanoparticles have been found to enhance their effect. AuNPs synthesized from the fungi, *T. viride* attached to vancomycin showed suppression of growth in vancomycin resistant *S. aureus* and *E. coli*, due to the proposed binding of vancomycin-AuNPs to the *S. aureus* transpeptidase, in place of terminal peptides of the glycopeptidyl precursors and easy transport across membrane in case of *E. coli*, leading to cell-wall lysis (Fayaz et al., 2011). Loading of multiple drugs such as ciprofloxacin, gentamycin, vancomycin and rifampicin on AuNPs, biogenically synthesized from *B. subtilis* exhibited growth suppression in *S. haemolyticus* and *S. epidermidis* due to enhanced surface area provided by the NPs for the drugs to bind (Roshmi et al., 2015). From the above examples, it is interesting to observe that the nanoparticles synthesized using the extracts of one microorganism are effective in the killing other microbial species and enhances the activity of existing antibiotics to overcome antimicrobial resistance phenotypes.

### Anti-biofilm Agents

The increasing incidences of antibiotic resistance are a major challenge in the area of antibiotic/antimicrobial development. An important reason for bacterial infection and their multidrug-resistant phenotype arises from the ability of the organism to form biofilms which make them resistant to drugs. Microbes such as *Staphylococcus aureus, Acinetobacter baumannii, Escherichia coli, Pseudomonas aeruginosa*, are known to cause opportunistic infections due to biofilm formation and thus, inhibiting it is a significant aspect explored in case of biogenic nanoparticles. Additionally, biomedical and dental devices are at high-risk of transmitting infections due to biofilm formation and nanoparticle coating has been examined as an effective option to avoid this. In most studies, the biofilm formation is generally assessed by cell staining (by crystal violet) and absorbance measurements or by observation under electron microscopes. In one research, TiO_2_ nanoparticles were synthesized utilizing *Bacillus subtilis* biomass. Afterwards, microbe-rich pond water was used for the growth of biofilm in solution or on glass slides along with the nanoparticles followed by irradiation of polychromatic light; the TiO_2_ nanoparticles acted as a photocatalyst releasing H_2_O_2_ to inhibit the biofilm growth (Dhandapani et al., 2012). Another early investigation, synthesized microbial Se and Te nanoparticles from the intracellular extracts of *Stenotrophomonas maltophilia* SeITE02 and *Ochrobactrum* sp. MPV1, which displayed distinct antimicrobial and anti-biofilm capabilities against both planktonic cells and biofilm cells of *E. coli* JM109, *S. aureus* ATCC 25923, and *P. aeruginosa* PAO1 with production of ROS suggested as the possible mechanism (Zonaro et al., 2015). The disinfectant properties of silver nanoparticles are pretty well-known. Silver nanoparticles harvested intracellularly from *B. licheniformis* biomass exhibited 90% anti-biofilm activity against *P. aeruginosa* and *S. epidermidis* (Kalishwaralal et al., 2010). Additionally, gold-silver bimetallic nanoparticles biogenically synthesized using the γ-proteobacterium *Shewanella oneidensis* MR-1, showed antimicrobial properties and were able to inhibit biofilm growth of *P. aeruginosa, S. aureus, E. coli*, and *Enterococcus faecalis* cultures at a concentration of 250 μM (Ramasamy and Lee, 2016). Fungi such as *Phanerochaete chrysosporium* have also showed promising biofilm eradication capability. Silver nanoparticles (~45 nm diameter) obtained from the extracellular extracts of the fungus were able to act on *E. coli* and *C. albicans*, even though the cell wall of both the strains are different (Estevez et al., 2020). An interesting negative effect of biofilm formation is observable in membranes, mostly used for wastewater treatment, where biofouling caused by microbial consortia present in the wastewater slurry, reduces the efficacy of the bioreactor. Microbial silver nanoparticles (bio-Ag0) of around 11 nm size, synthesized by *Lactobacillus fermentum* LMG 8900 were embedded in polyethersulfone (PES) membranes, and were further tested on (*E. coli* and *P. aeruginosa*) and another mixed culture in an activated sludge bioreactor. The membranes showed remarkable antibacterial and anti-biofilm activity in both cases over a test period of 9 weeks (Zhang et al., 2011). All the above instances reveal an excellent potential of microbial nanoparticles in inhibition and eradication of biofilm formation.

### Drug-Delivery Agents

Biogenic nanoparticles are important candidates over conventional ones as drug delivery agents due to their stability, biocompatibility, bioavailability, controlled drug release characteristics, targeted delivery and non-toxic nature. Such nano-agents can include nanospeheres, water soluble polymers, emulsions, micelles, and liposomes (Meng et al., 2010; Srivastava et al., 2021). As drug- carriers, what is needed is the ability to encapsulate a particular drug and release it conditionally at the disease site. Moreover, delivery agents should be able to cross the blood-tissue and cellular barriers for inter and intracellular transport in order to achieve targeted delivery of the drug-load at site (Fariq et al., 2017). However, it is pertinent to assess their safety to normal cells and efficacy in cancer cells at the very outset. Magnetotactic bacteria are known to convert magnetic greigite Fe_3_S_4_ and/or magnetite Fe_3_O_4_ into bilayer membrane bound structures known as magnetosomes, which can be used to encapsulate and carry drugs (Vargas et al., 2018; Ahmad et al., 2019). Bacterial magnetosomes loaded with doxorubicin were tested on H22 tumor-bearing mice and showed higher tumor suppression than doxorubicin alone (Sun et al., 2009). Magnetosomes from *Magnetospirillum gryphiswaldense* loaded with anti-4-1BB agonistic antibody have been used as immunotherapy against cancer in TC-1 mouse models (Tang et al., 2019). Taxol conjugated to gold nanoparticles obtained from the fungus *Humicola* sp. has been used for anti-tumor drug-delivery applications (Khan et al., 2014). Biogenic gold nanoparticles functionalized with moieties such as transferrin also hold potential to cross the blood-brain barrier to target drugs into the brain (Tripathi et al., 2015).

### Anti-cancer Agents

As an extension to the above section, pristine biosynthesized nanoparticles, without drug load have also been extensively used to develop anti-cancer agents. Platinum nanoparticles *Saccharomyces boulardii* were found to be effective against A431 epidermoid carcinoma and MCF-7 breast cancer cell lines (Borse et al., 2015). Gold nanoparticles biosynthesized from *Streptomyces cyaneus* exhibited anticancer activity *in vitro* against HEPG-2 human liver cancer cells and MCF-7 breast cancer cells, respectively. The plausible mechanism of action of the nanoparticles is through mitochondrial apoptosis, DNA impairment and induced detention of cytokinesis (El-Batal et al., 2015). Silver nanoparticles synthesized from the water extract of the endophytic fungi, *Cladosporium perangustum* has been found to reduce the viability of the MCF-7 cells through enhancement in the levels of caspase-3, caspase-7, caspase-8, and caspase-9 expression (Govindappa et al., 2020). Biocompatible terbium oxide nanoparticles synthesized using the biomass of fungus *Fusarium oxysporum* were effective in dose-dependent cytotoxicity in MG-63 and Saos-2 cell-lines while being non-toxic to primary osteoblasts; ROS production was enhanced and apoptosis was confirmed with nanoparticle treatment (Iram et al., 2016). ZnO nanoparticles biosynthesized from *Rhodococcus pyridinivorans*, loaded with anthraquinone showed cell-death in HT-29 colon carcinoma cells as compared to normal cells, and can thus find application as an anti-cancer agent (Kundu et al., 2014). AuNPs obtained from the fungi *Helminthosporium solani* conjugated to doxorubicin had higher uptake and comparable cytotoxicity in HEK293 cells compared to doxorubicin alone (Kumar et al., 2008). Similar gold and gadolinium oxide nanoparticles *Humicola* sp. could be conjugated to taxol or doxorubicin for anti-cancer applications (Syed et al., 2013; Khan et al., 2014). One interesting study used biomineralised magnetic nanoparticles (from magnetotactic bacteria), guided by MRI to convert the energy of near-infrared light into heat thus resulting in ablation of tumor cells with no-known toxicity. This was termed as a photothermal effect and the bacterial nanoparticles acted as a theranostic (therapy + diagnostic) in this case (Chen et al., 2016). Several *in-vivo* studies have revealed the potential of bacterial magnetic nanoparticles. In another study, BALB/C mouse were immunized with bacterial magnetosomes to observe their immune response, and found to have not so significant response, proving their drug delivery potential (Meng et al., 2010).

### Diagnostics and Imaging Agents

In general, nanoparticles find increasing applications in diagnostics and as biosensors often conjugated to diagnostic enzymes (Rossi et al., 2004; Ghosh et al., 2018a,b). In recent times, biogenic nanoparticles have also been explored as biosensors and in imaging modalities such as MRI. In MRI, contrast agents comprising of magnetites are found to be synthesized by several Gram negative magnetotactic bacteria (MTB) in the form of magnetosomes, which are intracellular organelles with a lipid bilayer enclosing crystals of magnetic iron oxides (Uebe and Schuler, 2016). Bacterial magnetosomes display higher r2 relaxivity than synthesized nanoparticles and have shown application in targeting human epidermal growth factor receptor-2 (HER2) expressing tumor cells. Relaxivity is a measure of how sensitive a contrast agent is. For similar compounds, a molecule with higher relaxivity would provide equivalent contrast at a lower dose compared to a low relaxivity compound. A lower dose may lower the risk of the nanoparticle toxicity (Jacques et al., 2010). In orthotopic breast cancer models, intravenous administration of HER2-targeting bacterial magnetosomes, showed augmented contrast in the MR signals (Zhang Y. et al., 2018). Another study created RGD-peptide expressing magnetosomes by generic engineering *Magnetospirillum magneticum* AMB-1 strain, which targeted αvβ3 integrins-overexpressing brain tumor cells in gliomas as evident in MRI (Boucher et al., 2007, 2017; Zhao, 2017). A theranostic photothermal therapy of cancer using magnetic nanoparticles of the same bacterial strain under the guidance of MRI was achieved *in vitro* and *in vivo* by another group (Chen et al., 2016). An interesting study employed magneto-endosymbionts as living contrast agent in the iPSC-derived cardiomyocytes, which could be tracked by MRI and cleared out within 1 week, thus enhancing biocompatibility (Mahmoudi et al., 2016). Bacteriogenic metal nanoparticles such as that of copper, palladium and gold have also been explored for their potential in biosensing (Rai et al., 2016; Ghosh, 2018). In an interesting study, AuNPs synthesized from *Candida albicans* were conjugated to liver cancer cell surface specific antibodies. Thus, when used to probe into liver cancer cells, they could uniquely bind to the liver cancer specific surface antigen, thus distinguishing them from normal cells. Such nanoparticles could thus find application as a diagnostic or as a carrier of anti-cancer drugs (Chauhan et al., 2011).

### Other Medical Uses

As is evident, microbial synthesized nanoparticles find more than the above stated pharmaceutical applications. One early study employed the biomass of *Brevibacterium casei* to reduce AgNO_3_ and HAuCl_4_ to obtain silver and gold nanoparticles from the intracellular extracts, which were further explored as an anti-coagulant of human plasma (Kalishwaralal et al., 2010). From fungal species, gold nanoparticles derived from *Nigrospora oryzae* displayed anthelmintic activity (paralysis and death) against the cestode parasite *Raillietina* sp. (Kar et al., 2014). Antimicrobial carbon dots (CDs) were synthesized by hydrothermal method from cell free supernatant of *Lactobacillus acidophilus* and they showed antimicrobial activity against *Escherichia coli* (Gram-negative) and *Listeria monocytogenes* (Gram-positive) (Kousheh et al., 2020). Nanocellulose is another nanoscale material which is predominantly synthesized by bacteria. Scaffolds based on nanocellulose (NC) have pivotal applications in tissue engineering (TE) like to repair, improve or replace damaged tissues and organs, including skin, blood vessel, nerve, skeletal muscle, heart, liver, and ophthalmology, mainly due to the biocompatibility, water absorption, water retention, optical transparency, and chemo-mechanical properties (Luo et al., 2019). Some of these nanocelluloses has been clinically approved and available in the market in the form of patents for wound healing, burn treatment and cosmetic applications (Brown et al., 2015).

## Conclusion and Future Prospects

Nanoparticles synthesized by microbes prove promising for several biomedical and therapeutic applications due to their controlled biocompatible dimensions and unique properties. Methods of biosynthesis are also beneficial since nanoparticles are often coated with a lipid layer/biomolecules that gives physiological solubility and stability, which is essential for biomedical applications and is the bottleneck of other synthetic methods. However, biogenic nanoparticles pose a few challenges which need to be addressed for large scale applications. Till now, the lack of monodispersity, time intensive production process, low production rates, and batch to batch variations has limited their use on commercial scale. There are some important aspects which might be considered in the process of synthesis of well-characterized nanoparticles. For the synthesis of highly stable and well-characterized NPs, biological protocols may be used when critical aspects such as organism types, inheritable and genetic properties of organisms, optimal conditions for cell growth and enzyme activity, optimal reaction conditions, and biocatalyst state selection have been considered. Additionally, most biomedical studies with microbial nanoparticles have been accomplished *in-vitro* and large scale clinical trials and safety tests are of utmost importance to realize their effects *in-vivo*. Thus, with further in-depth studies, it is hoped that microbial nanoparticles will hold immense potential in medicine and healthcare.

## Author Contributions

SG and RA conceptualized and prepared the manuscript. SK has critically reviewed the manuscript. MZ helped in critically assessing the manuscript and addressing the review comments with inputs which were further included in the revised manuscript. All authors contributed to the article and approved the submitted version.

## Conflict of Interest

The authors declare that the research was conducted in the absence of any commercial or financial relationships that could be construed as a potential conflict of interest.
